# taVNS as a potential countermeasure for neurocognitive decline in microgravity

**DOI:** 10.3389/fnins.2025.1724872

**Published:** 2025-11-27

**Authors:** L. Fricke, C. Wienke, T. Zaehle

**Affiliations:** 1 Medical Faculty, Institute of Medical Psychology, Otto-von-Guericke University of Magdeburg, Magdeburg, Germany; 2 Research Group “Magdeburger Arbeitsgemeinschaft für Forschung unter Raumfahrt- und Schwerelosigkeitsbedingungen” (MARS), Otto-von-Guericke-University of Magdeburg, Magdeburg, Germany; 3 Department of Neurology, Medical Faculty, Otto-von-Guericke University of Magdeburg, Magdeburg, Germany

**Keywords:** taVNS, cognitive impairments, microgravity, parabolic flight, space medicine

## Abstract

Exposure to microgravity induces significant physiological, cognitive, and psychomotor changes in the human body. While countermeasures such as resistance exercise and cardiovascular conditioning have been developed to address musculoskeletal and circulatory issues, there remains a critical gap in mitigating neurophysiological and cognitive deficits caused by microgravity. Transcutaneous auricular vagus nerve stimulation (taVNS) is a non-invasive neuromodulation technique that promises to enhance psychomotor function, and cognitive performance in microgravity as well as on Earth. This article examines the challenges of spaceflight, particularly cognitive impairments and related psychomotor dysfunction, and explores the potential application of taVNS in space. The neurophysiological mechanisms underlying microgravity-related decline and the proposed mechanism of action of taVNS are discussed, focusing on its effects on neuroplasticity, autonomic regulation, and sensorimotor integration. taVNS emerges as a promising countermeasure to mitigate neuropsychological impairments associated with exposure to microgravity.

## 1 Introduction – operating in an extreme environment

Astronauts operating in space are subjected to a unique constellation of physical and psychological stressors that can compromise both physiological integrity and cognitive performance (Baker et al., 2019; Clark et al., 2019; Marazziti et al., 2022). Social isolation, confinement, and exposure to a novel and high-risk environment can lead to significant psychological stress responses, despite extensive pre-mission training (Kanas, 2023; Oluwafemi et al., 2021; Yin et al., 2023). These conditions have been linked to increased risk of anxiety, paranoia, and emotional dysregulation (Marazziti et al., 2022), likely mediated by dysregulations of the hypothalamic–pituitary–adrenal (HPA) axis and sustained elevations in cortisol (Yin et al., 2023). Circadian disruption, driven by artificial lighting and the absence of natural light–dark cycles aboard spacecraft further contributes to sleep disturbances, cognitive impairments, and mood instability (Marazziti et al., 2022). The combined effects of chronic sleep restriction, − ambient noise and aerratic light exposure, are associated with further impairments of overall cognitive performance including deficits in memory, decision-making, and attention (Alhola and Polo-Kantola, 2007; Flynn-Evans et al., 2020; Piltch et al., 2025), as well as neuroinflammatory processes that additionally undermine executive function and emotional regulation (Yin et al., 2023).

These psychologically mediated stressors are exacerbated by immediate and direct neurocognitive consequences of microgravity exposure. In contrast to the gradual onset of psychological dysregulation, cognitive and psychomotor impairments occur immediately upon exposure to microgravity and have become increasingly apparent in the context of spaceflight and long-duration missions aboard the International Space Station (Manzey and Lorenz, 1998; Strangman et al., 2021).

Microgravity leads to measurable decrements in core cognitive domains, including attention, memory, and executive functions. Attention deficits—manifesting as reduced vigilance and impaired situational awareness – seem to be linked to altered prefrontal cortex activity (Strangman et al., 2014; Basner et al., 2021). Memory functions, particularly working memory and time-constrained long-term memory retrieval—also deteriorates under microgravity analog conditions (Koppelmans et al., 2015). Executive functions such as planning, cognitive flexibility, and problem-solving are similarly compromised under microgravity exposure (Manzey and Lorenz, 1998; Strangman et al., 2021).

Findings from in space-analog environments such as NASA’s Human Exploration Research Analog (HERA) further substantiate these deficits, revealing reductions in cognitive flexibility and slowed response times [Psychomotor Vigilance Test (PVT)], Emotion Recognition Task, Digit Symbol Substitution Test (DSST) under simulated mission conditions (Basner et al., 2021; Basner et al., 2015; Roy-O'Reilly et al., 2021).

The cognitive deficits can pose a serious risk to mission-critical operations, particularly during extravehicular activities and emergency responses (Barger et al., 2014). Maintaining terrestrial levels of cognitive and psychomotor performance is thus essential for preserving work capacity, situational awareness, decision-making competence, and psychological resilience during space missions and must be protected proactively. In light of planned long-duration missions beyond low Earth orbit, these impairments highlight the urgent need for proactive—rather than reactive—countermeasures. Preventive strategies are likely to be more effective in preserving astronaut performance and well-being over time. Accordingly, the identification and validation of innovative and practical countermeasures should become a research priority.

Current countermeasures for cognitive and psychomotor decline in spaceflight include behavioral adaptation (e.g., multitasking and sensory reweighting exercises), cognitive training via in-flight test batteries, and optimized sleep–wake scheduling (Tays et al., 2021; Roy-O'Reilly et al., 2021). Pharmacological interventions, such as psychostimulants like modafinil, are occasionally used to sustain alertness during extended wakefulness (Blue et al., 2019; Wu et al., 2018). Additional strategies include non-pharmacological sensorimotor technologies (e.g., galvanic vestibular stimulation), virtual reality-based stress management, and emerging neurostimulation techniques such as transcranial magnetic stimulation (Khalid et al., 2023; Sharp et al., 2025).

A promising potential countermeasure, especially against cognitive impairments, is transcutaneous auricular vagus nerve stimulation (taVNS). As a non-invasive, portable, and low-risk method, taVNS has shown efficacy in mitigating cognitive deficits across a range of Earth-based conditions, including chronic fatigue syndrome (Gierthmuehlen et al., 2025), cognitive and motoric stroke rehabilitation (Chen et al., 2024; Wang et al., 2023), and age-related cognitive decline (Cibulcova et al., 2024). Its practical usability and physiological compatibility render it particularly appealing for application in spaceflight.

This article proposes taVNS as a novel neuromodulatory countermeasure to support cognitive resilience during space missions. We review the neurocognitive challenges posed by microgravity, summarize existing evidence for the cognitive effects of taVNS, and outline its potential implementation within operational space medicine.

## 2 Microgravity-induced cognitive and psychomotor challenges

While psychological stressors accumulate gradually, cognitive and sensorimotor disruptions emerge immediately upon entry into microgravity (Manzey and Lorenz, 1998; Strangman et al., 2021). These include declines in attention, cognitive flexibility, visuo-motor coordination, and fine motor precision (Manzey and Lorenz, 1998; Strangman et al., 2021). Such deficits threaten operational safety, particularly during extravehicular activities and technical emergencies (Barger et al., 2014).

Seminal work by Manzey and Lorenz (1998) systematically summarized cognitive performance data from short and longterm space missions. Their findings revealed microgravity-induced impairments, notably in selective and divided attention, and dual-task performance. Deficits were particularly evident in visuo-motor tracking tasks and multitasking scenarios, indicating that microgravity disrupts the integration of perceptual and executive control processes—a combination essential for operational performance (Manzey and Lorenz, 1998).

Recent investigations have further refined our understanding of microgravity’s impact on attention. In this study using a modified Posner cueing paradigm, Salatino et al. (2021) demonstrated that microgravity amplifies stimulus-driven (exogenous) attentional capture by peripheral cues, while concurrently impairing the voluntary (endogenous) maintenance of attention to centrally cued targets. This attentional reweighting suggests a shift toward bottom-up processing in 0 g, likely driven by the transient unweighting of otolithic input and increased dependence on visual saliency (Salatino et al., 2021).

Friedl-Werner et al. (2021) provided complementary evidence from a Go/No-Go continuous performance task administered across 31 parabolas. Participants exhibited performance deterioration beginning with the first exposure to weightlessness. Reaction times slowed and error rates increased with successive parabolas, indicating a cumulative attentional burden. However, these impairments returned to baseline after flight, highlighting the acute but reversible nature of microgravity-related deficits (Friedl-Werner et al., 2021).

Beyond the acute effects of short-term microgravity during parabolic flights, long-duration orbital spaceflight studies aboard the International Space Station (ISS) have revealed more persistent impairments, particularly in sensorimotor control and cognitive-motor integration. Tays et al. (2021) investigated 15 astronauts before, during, and after six-month ISS missions using a battery of cognitive and motor assessments. Participants showed impairments in balance, mobility, and fine motor control, including slower performance on the Purdue Pegboard and Functional Mobility Test. These findings suggest impairments in cognitive-motor integration, sensorimotor coordination, and processing speed, reflecting difficulties in the rapid translation of perceptual input into motor output—particularly in tasks requiring simultaneous planning, attention shifting, and fine motor execution. While core cognitive functions such as working memory and dual-tasking remained largely intact, there were subtle signs of slowed information processing and degraded coordination were observed in tasks involving combined motor and cognitive demands (Tays et al., 2021). Sensorimotor performance has also been shown to decrease during parabolic flights, without signs of intra-flight recovery (Bock et al., 2003; Schneider et al., 2009). Bock et al. (2003) found that single-task performance declined by roughly 50% during microgravity, accompanied by transient increases in dual-task interference. These effects were interpreted as reflecting short-term sensorimotor adaptation rather than lasting impairment, and are not solely attributable to distraction or fatigue. Neurophysiological evidence suggests that transient disruptions in frontoparietal network function underlie the observed deficits in visuomotor coordination and attentional control (Salatino et al., 2021).

Interestingly, Wollseiffen et al. (2016) reported the opposite pattern, observing faster reaction times and stable accuracy during mental arithmetic in both novice and experienced flyers. The authors suggested that these effects likely reflect stress-related arousal or adaptation rather than a facilitative or impairing effect of microgravity on cognition.

Taken together, although some studies have reported improved performance under microgravity, the majority of empirical evidence still supports the view that weightlessness compromises attentional control and cognitive-psychomotor coordination, underscoring the need for further systematic investigation into the neurocognitive and autonomic mechanisms underlying microgravity-induced performance changes.

### 2.1 Brain network disruption and structural plasticity

Emerging evidence from neuroimaging studies indicates gravitational changes can—even by single parabolic flight—can rapidly alter brain connectivity. In a landmark study, van Ombergen et al. (2017) demonstrated significant decrease in functional connectivity within the right temporoparietal junction (rTPJ)—a key hub for multisensory integration and spatial awareness—after a single parabolic flight. This effect remained after controlling for pharmacological confounds using a scopolamine-treated control group. These findings highlight that brief alterations in gravitational loading are sufficient to perturb core vestibular-cognitive circuits (van Ombergen et al., 2017; van Ombergen et al., 2017).

Although it remains unclear whether gravitationally induced neurophysiological changes reflect maladaptive dysfunction or compensatory plasticity, there is growing evidence that both direct gravitational effects and indirect stress-related mechanisms jointly contribute to the cognitive and neural disturbances observed during spaceflight (Schneider et al., 2009). These two pathways—gravitational unloading of vestibular and somatosensory systems, and psychosocial or physiological stressors—may interactively disrupt functional brain networks, particularly those involved in spatial orientation, multisensory integration, and executive control (Schneider et al., 2009).

When behavioral, neurochemical, and neuroimaging findings are considered in concert, a more comprehensive picture emerges: microgravity alters not only cognition and perception, but also the structure of the brain itself, possibly due to a rapid adaptation to weightlessness. Multiple MRI studies have documented robust neuroanatomical changes particularly following long-duration spaceflight, including upward brain displacement within the skull, ventricular enlargement, alterations in white matter tracts, and redistribution of cerebrospinal fluid (CSF),- presumably as a compensatory response to fluid shifts and intracranial pressure changes under weightlessness (Demertzi et al., 2016; Roberts et al., 2017; van Ombergen et al., 2019; Lee et al., 2019). In addition, post-flight MRI studies of ISS astronauts have revealed cortical thickening in motor regions. This effect is thought to reflect long-term neuroplastic adaptations to the unusual motor demands in microgravity, where posture-controlling muscles are largely relieved of gravitational load because they no longer need to work against gravity (Roberts et al., 2017). While these findings underscore the brain’s plasticity, they also raise critical concerns about the functional costs of prolonged exposure to microgravity and the need for protective strategies.

Microgravity-induced cognitive impairments in attention, executive functioning, and spatial working memory can be linked to structural and functional brain alterations. Specifically, changes in ventricular enlargement, brain shift, and white matter integrity may compromise connectivity within fronto-parietal and prefrontal circuits—networks critical for top-down control, goal-directed behavior, and adaptive motor planning (Yuan et al., 2018; Lee et al., 2019; Roberts et al., 2017).

Taken together, these insights underline the urgent need for targeted countermeasures to preserve cognitive integrity and operational capacity during future space exploration.

## 3 taVNS as potential countermeasure

In the context of crewed spaceflight missions, there is a high demand for non-invasive, practical methods to stabilize cognitive and psychomotor performance. Transcutaneous auricular vagus nerve stimulation (taVNS) represents a promising option, which has already proven effective under terrestrial conditions across a wide range of domains, including neurorehabilitation, fatigue syndromes, and cognitive aging, and is considered a safe and well-tolerated intervention (Kim et al., 2022; Redgrave et al., 2018).

taVNS is a non-invasive neuromodulatory technique that applies weak, electric pulses to the auricular branch of the vagus nerve via electrodes placed on the ear—typically at the cymba conchae or tragus (Badran et al., 2018; Frangos et al., 2015; Ludwig et al., 2021; Wienke et al., 2023). Stimulation trains of single pulses of 200–250 μs are applied at frequencies typically between 20 and 30 Hz with the stimulation intensity adjusted to the individual pain or perception threshold (Ludwig et al., 2021).

This stimulation activates various neurophysiological networks via the nucleus tractus solitarius (NTS) in the brainstem, including the locus coeruleus (LC), the nucleus parabrachialis, the hypothalamus, and cortical areas of the limbic and prefrontal systems (Badran et al., 2018; Frangos et al., 2015). The LC is the brain’s primary source of noradrenergic projections and plays a key role in regulating attention, vigilance, and cognitive flexibility (Kurniawan et al., 2021; Sara, 2009). Recent evidence suggests that taVNS modulates the LC-noradrenaline system via activation of the NTS, thereby supporting adaptive cognitive processes (Butt et al., 2020; Maier and Grueschow, 2021). Animal studies have shown that vagal stimulation increases neuronal activity in the LC and noradrenaline levels in the prefrontal cortex (Hulsey et al., 2017; Raedt et al., 2011). Human studies also provide evidence that taVNS modulates noradrenergic arousal, detectable via pupillometric indicators (e.g., pupil dilation) and changes in frontal theta oscillations in EEG (Keute et al., 2020; Sharon et al., 2021; Wienke et al., 2023).

On a functional level, taVNS modulates central mechanisms of attentional control and executive function. Due to its good tolerability, ease of use, and technical robustness, taVNS is also considered a promising intervention to support neurocognitive processes under physiological or psychological stress (Badran et al., 2018; Frangos et al., 2015; Kim et al., 2022; Le Roy et al., 2023; Zhao et al., 2023).

To ensure that observed effects are not merely caused by the sensory perception of the stimulation, empirical taVNS studies typically employ a sham stimulation as placebo condition. For this, stimulation is applied to regions of the auricle that are free of vagal fibers and should therefore not affect the vagal afferent pathway (Butt et al., 2020). In most cases, this is the earlobe (Farmer et al., 2020; Butt et al., 2020) but due to recent concerns about its suitability (Borges et al., 2021; Rangon, 2018) the scapha, i.e., the upper part of the auricle, has been proposed as a potentially better suited sham location (Cakmak et al., 2018).

Numerous controlled studies have shown significant improvements in executive functions, reaction speed, and conflict processing—both in healthy individuals and in clinical populations (Burger et al., 2020; Fischer et al., 2018; Ridgewell et al., 2021). A meta-analysis of 19 studies reported consistent effects on the accuracy of cognitive performance, particularly in executive functions (Ridgewell et al., 2021). Even under specific stressors such as sleep deprivation, taVNS has shown positive effects on working memory (Le Roy et al., 2023; Zhao et al., 2023).

A recent randomized controlled trial by Cibulcova et al. (2024) adds further support to the cognitive benefits of taVNS. In this study, 76 healthy adults underwent a 2-week course of daily taVNS (4 h/day), compared to a sham stimulation group. The researchers found significant improvements in immediate recall and short-term memory in the active taVNS group, with effects persisting at follow-up. If confirmed under microgravity conditions, such protocols used in long-term missions to sustain cognitive performance over time and prevent performance decline due to cumulative stress, fatigue, or environmental strain.

Wienke et al. (2023) showed that phasically applied taVNS impulses significantly modulate attentional performance, reflected in both pupillary responses and EEG oscillations—especially in lower frequency ranges. Rufener et al. (2023) similarly demonstrated that taVNS alters cortical processing and perception of auditory stimuli, emphasizing its role in sensory-cognitive integration. taVNS is also being discussed as a potential method for reducing subjective fatigue and enhancing cognitive resilience in everyday life (Linnhoff et al., 2023).

Systematic reviews (Beste et al., 2016; Ventura-Bort et al., 2025; Giraudier et al., 2022) emphasize that taVNS shows consistent effects on neurocognitive functions across a wide range of experimental studies—particularly in executive control, inhibitory processing, and emotional regulation. The authors highlight the robustness of taVNS effects across paradigms, supporting the assumption that the method could be effective even under exceptional conditions such as microgravity (Giraudier et al., 2022; Ventura-Bort et al., 2025).

Additionally, taVNS is a potential strategy to mitigate stress-related cognitive impairments. Activity of the NTS can influence the release of corticotropin-releasing factor (CRF) in the paraventricular nucleus (PVN) of the hypothalamus, stimulating the pituitary gland to secrete ACTH and ultimately triggering the release of glucocorticoids (cortisol) from the adrenal glands. This pathway modulates the activity of the HPA axis in response to stress (Trevizan-Baú and McAllen, 2024).

Beyond these benefits, taVNS also influences autonomic nervous system regulation, which has significant implications for astronaut well-being (de Couck et al., 2017; Buschman et al., 2006; Clancy et al., 2014). Chronic stress, sleep deprivation, and social isolation are common in spaceflight and have been linked to cognitive impairments and mood disturbances (Flynn-Evans et al., 2020). Studies have shown that taVNS modulates autonomic activity, promoting stress resilience, emotional regulation, and improved sleep efficiency (Hein et al., 2013; Le Roy et al., 2023). By stabilizing these physiological processes, taVNS could mitigate secondary cognitive impairments arising from stress-related dysregulation, thereby enhancing overall astronaut performance and psychological well-being during extended missions.

In contrast to other proposed electrical countermeasures such as galvanic vestibular stimulation (GVS; 74), taVNS targets a distinct afferent pathway and functional mechanism. GVS delivers weak bipolar currents via electrodes placed over the mastoid processes to modulate vestibular nerve activity, thereby inducing artificial motion cues, postural reflexes, and oculomotor responses. This approach has been proposed as a tool to mitigate space motion sickness, spatial disorientation, and balance disturbances in microgravity by providing surrogate gravitational information to the vestibular system (Soto and Vega, 2024; Marchand et al., 2025). Rather than directly restoring vestibular input, taVNS aims to stabilize sensory—cognitive processing, vigilance, and stress resilience, domains repeatedly challenged during prolonged missions. From an operational perspective, the two technologies address complementary targets: GVS primarily supports vestibular adaptation and spatial orientation, whereas taVNS primarily supports cognitive–autonomic regulation and performance under sustained demand. Both approaches are therefore not mutually exclusive and could, in principle, be combined in integrated hardware solutions to jointly support posture, gaze control, and higher-order cognitive function in microgravity.

Taken together, these findings suggest that taVNS offers a promising, low-risk, and technically viable countermeasure to mitigate microgravity-induced cognitive decline. While the neurocognitive risks associated with spaceflight are well established, systematic investigations of taVNS under microgravity conditions are still lacking. Given its safety, versatility, and evidence base, taVNS warrants serious consideration as an operational tool to enhance astronaut performance and well-being during future long-duration missions.

## 4 Parabolic flight setting

Parabolic flight provides a unique and experimentally accessible platform for investigating human neurocognitive function under true microgravity conditions. By generating repeated 20–25 s phases of weightlessness within a controlled flight profile, parabolic flights allow real-time access to participants and measurement systems during microgravity exposure—unlike actual space missions, where *in situ* cognitive testing is logistically constrained (Shelhamer, 2016; Montag et al., 2016). As such, parabolic flight remains the most efficient and methodologically versatile means of simulating microgravity on Earth. This setting enables the precise temporal mapping of cognitive and physiological changes across distinct gravitational phases. By implementing repeated measurements—before, during, and after 0 g epochs—researchers can capture the immediate and residual effects of interventions such as taVNS and relate them systematically to behavioral outcomes such as reaction time, error rate, and vigilance fluctuations. Beyond its logistical advantages, the parabolic flight environment offers high ecological validity for studying early adaptation processes to weightlessness. Empirical evidence shows that even short-term exposure to microgravity during parabolic flight measurably impairs cognitive performance, including attention, response selection, and executive control. These effects can be objectively quantified using standardized neuropsychological assessments, making healthy volunteers viable models for probing microgravity-induced dysfunction.

Importantly, the parabolic flight setting combines experimental controllability, physiological relevance, and methodological efficiency—qualities that render it uniquely suited for the initial evaluation of taVNS as a cognitive countermeasure. It allows for the fine-grained testing of stimulation protocols, dose – response relationships, and latency of effect, all within the operational constraints likely to apply in future spaceflight deployments.

**Figure 1:**
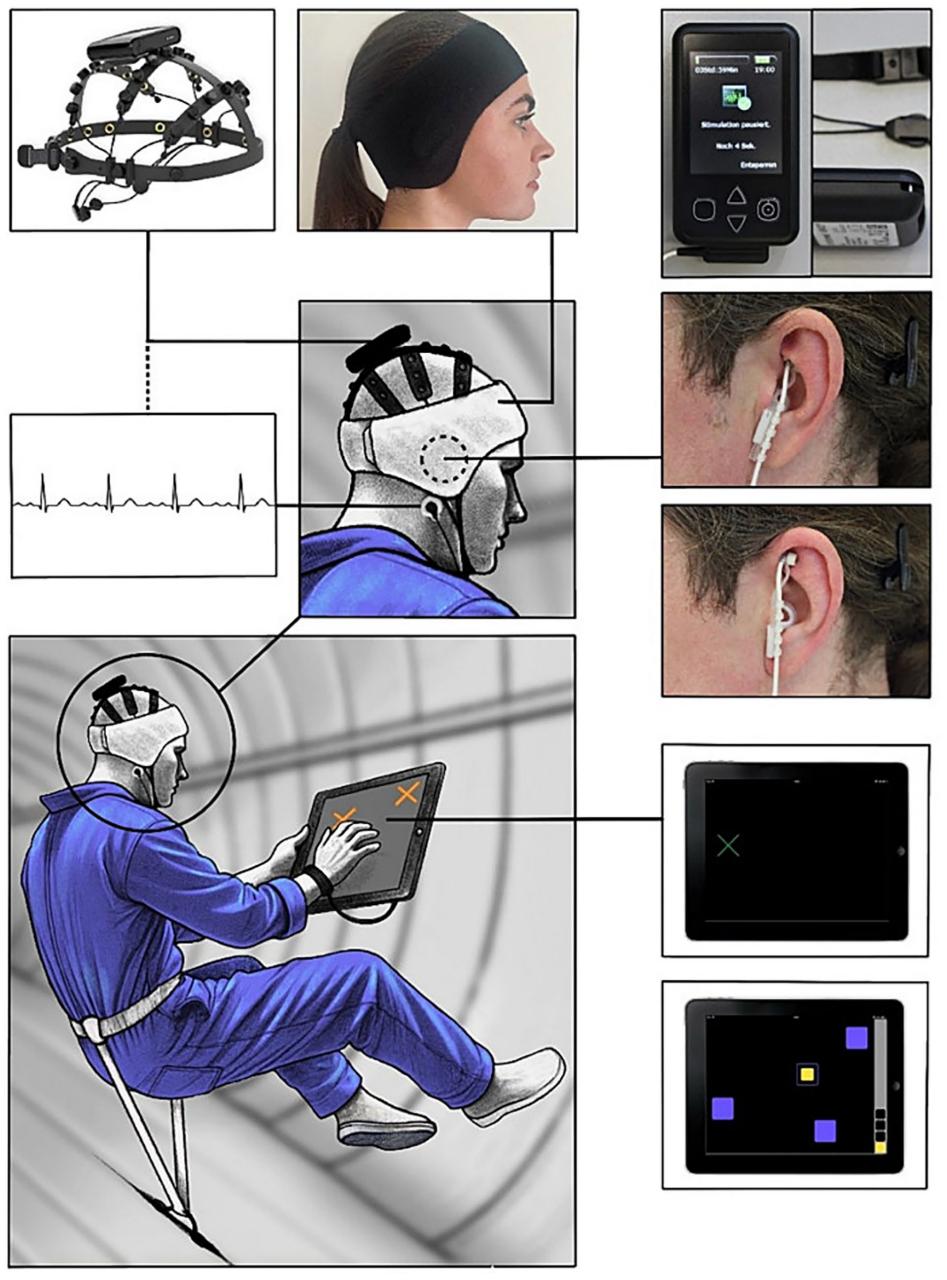
Conceptual setup in a parabolic flight study. Central illustration (bottom left) shows a participant in a parabolic flight setting, secured with a harness, performing cognitive tasks on a tablet while floating in microgravity. The participant wears an EEG/ECG cap, auricular electrodes for taVNS stimulation and a fixating head band. The surrounding panels display the individual components of the setup: the wireless, portable and liquid-free EEG/ECG system that represents a unique opportunity for use in space-like environments and which was originally designed as home recording device (Baum et al., 2022; Hinrichs et al., 2020), and its application on the head (top left, Telemedi, Magdeburg, Germany), the CE certified taVNS stimulator device (top right, Cerbomed Nemos^®^, tVNS Technologies Erlangen, Germany) and electrode placement (middle right) at the ear in verum (cymba concha region; upper panel) vs. sham (scapha region; lower panel) condition. At the lower right, screenshots of the tablet-based cognitive tests illustrate typical task displays (e.g., reaction time and working memory tasks).

Accordingly, we propose parabolic flight studies to provide the first targeted investigation of taVNS as a neuromodulatory intervention under simulated 0 g conditions. Such investigations could combine taVNS with wearable EEG systems to monitor neural activity patterns, attentional states, and cortical dynamics in real time, thereby providing mechanistic insight into how stimulation affects brain function under gravitational transitions. The employment of standardized cognitive test batteries will allow for an objective evaluation of domains such as executive functions and attention, based on parameters including reaction time, error rate, and psychomotor speed during alternating gravity phases (Figure 1).

This envisioned study should conduct randomized, sham-controlled trials during parabolic flights to systematically evaluate the feasibility and neuromodulatory potential of taVNS in microgravity. Participants should be randomly assigned to either verum stimulation or sham control to enable a comparison between active and placebo conditions. Assessments should be scheduled at three key time points: (i) a pre-flight baseline session on ground, (ii) testing during alternating 0 g and 1 g phases of the parabolic maneuvers, and (iii) a post-flight follow-up session. The baseline measurement serves to establish each participant’s individual reference level for cognitive and physiological parameters prior to microgravity exposure and stimulation. This enables within-subject comparisons to determine the magnitude and direction of changes relative to ground-level performance. During flight, repeated assessments across 0 g and 1 g phases will allow the differentiation between gravity-related performance fluctuations and stimulation-specific effects. The post-flight follow-up provides insight into short-term carryover or recovery effects once normal gravity has been re-established. Planned analyses will therefore focus on (a) within-subject contrasts across gravity phases (0 g vs. 1 g) and time points (pre-, intra-, post-flight), and (b) between-group comparisons (verum vs. sham). By integrating behavioral, electrophysiological, and autonomic measures, the study aims to identify whether taVNS modulates cognitive performance and neural dynamics specifically during microgravity, or whether effects persist beyond immediate exposure.

Ideally, a cognitive test battery targeting several distinct cognitive domains, including executive functions (cognitive flexibility, inhibitory control, working memory) and attentional processes (sustained and selective attention) should be used to assess objective performance parameters such as reaction time, error rate, accuracy, and psychomotor speed. In parallel, wearable EEG systems can provide continuous recordings of neural activity, enabling the characterization of cortical dynamics, oscillatory patterns, and attentional states in real time across gravitational phases. Finally, physiological markers, such as heart rate variability, can be considered to capture taVNS effects on autonomic regulation.

Such comprehensive study designs aim not only to assess feasibility under the unique conditions of parabolic flight but also to provide mechanistic insight into how vagal neuromodulation interacts with cognition and physiology in extreme environments. The resulting knowledge will form a crucial basis for future applications of taVNS in long-duration space missions.

A conceptual setup integrating these components is illustrated in Figure 1. As mentioned above, we propose wearable EEG acquisition, auricular stimulation, and tablet-based cognitive testing during parabolic flight. By integrating neurophysiological and behavioral measures, parabolic flight studies could establish whether taVNS meaningfully influences cognitive performance and operational readiness in acute microgravity. The resulting knowledge would inform stimulation protocols, timing strategies, and technical requirements for applying taVNS in more prolonged space missions.

Beyond its role as a feasibility and proof-of-concept study, the parabolic flight experiment might lay the foundation for the long-term vision of implementing taVNS aboard the International Space Station (ISS) and, eventually, on future deep space missions. In this scenario, taVNS could be used to support sustained cognitive stability, attentional control, and sensorimotor integration over extended periods of microgravity exposure. Given taVNS’s additional effects on autonomic tone, stress regulation, and potentially sleep quality—as reported in terrestrial applications—it may serve as a multifaceted tool to buffer against the cumulative physiological and psychological stressors of spaceflight.

### 4.1 Practical and engineering considerations for a space-ready taVNS system

Although clinically approved and research-graded taVNS devices are already available on Earth (e.g., CE-certified portable stimulators), their direct use in spaceflight is not straightforward and requires targeted adaptation. A space-ready taVNS system would need to (i) ensure robust and reproducible electrode contact at the cymba conchae or tragus under microgravity, (ii) provide stable constant-current stimulation within defined safety limits and continuous impedance monitoring, and (iii) comply with spacecraft-specific constraints regarding electromagnetic compatibility, radiation tolerance, materials, and hygiene.

In practical terms, such a system could likely be integrated into existing communication headsets or helmet liners, using ergonomically shaped auricular electrodes fabricated from biocompatible, low off-gassing materials. The stimulator unit would require rechargeable, mission-certified batteries, predefined stimulation protocols, and straightforward user control to allow safe operation during or prior to cognitively demanding tasks. A phased validation pathway is essential, beginning with ground-based usability and safety testing, followed by parabolic flight experiments and operational assessment in spaceflight analogs, before any on-orbit technology demonstration. These steps would ensure that taVNS as a countermeasure is not only physiologically plausible but also technically and operationally feasible within real mission constraints.

## 5 Conclusion

Prolonged space missions pose significant risks to astronaut health, particularly in cognitive function, psychomotor abilities. taVNS offers a non-invasive, evidence-based approach to counteract these challenges through its neuromodulatory effects on the autonomic and central nervous systems. Integrating taVNS into space medicine protocols or helmets represents a promising step toward enhancing astronaut performance and long-term well-being during deep-space exploration missions.

Current countermeasures for cognitive and psychomotor decline in spaceflight primarily focus on behavioral adaptation, cognitive training, and pharmacological interventions (Barratt et al., 2019). While these strategies have demonstrated efficacy, they present limitations in terms of feasibility, side effects, and long-term sustainability. Behavioral adaptations and cognitive training require extensive pre-mission preparation and continuous reinforcement, while pharmacological interventions carry risks of dependency, tolerance, and individual variability in response. In contrast taVNS offers a non-invasive neuromodulatory approach that could provide real-time cognitive stabilization and psychomotor enhancement without the need for prolonged adaptation (Burger et al., 2020; Clark et al., 2019; Redgrave et al., 2018).

**Figure 2:**
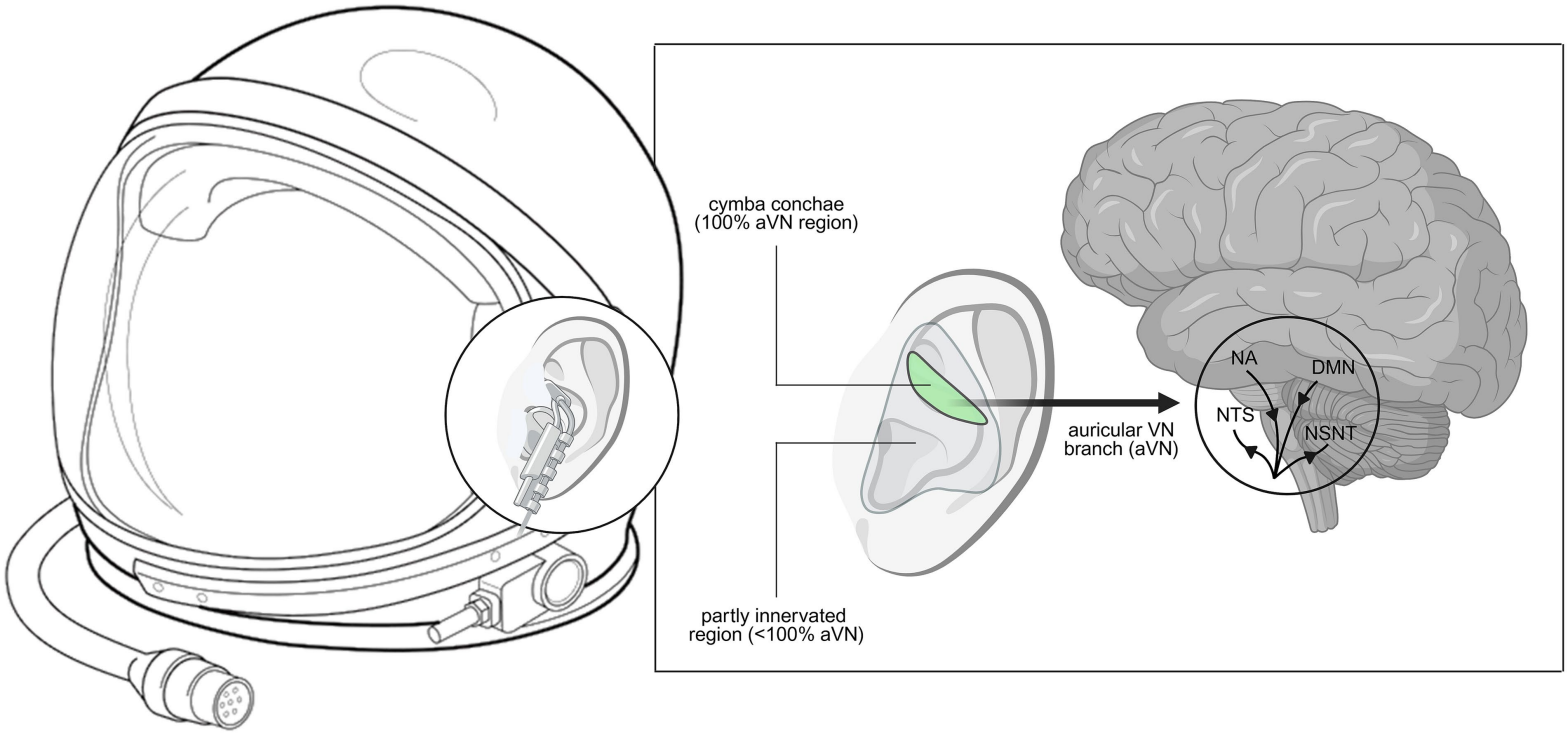
Conceptual integration of taVNS into astronaut helmet (adapted from Kaniusas et al., 2019). Conceptual integration of taVNS into an astronaut helmet. The left panel illustrates a schematic helmet design equipped with embedded auricular electrodes positioned at the cymba conchae for continuous stimulation during spaceflight. The inset highlights the electrode placement at the ear. The right panel depicts the underlying neurophysiological pathway: stimulation of the auricular branch of the vagus nerve activates afferent projections to the nucleus tractus solitarius (NTS) and related structures, with downstream influences on the locus coeruleus noradrenaline (NA) system, the default mode network (DMN), and broader cortical and subcortical targets. This pathway mediates effects on autonomic regulation, cognition, and stress resilience, ultimately linking peripheral stimulation to central nervous system processes relevant for astronaut performance. This vision illustrates how taVNS could be seamlessly integrated into standard space equipment, enabling real-time neuromodulation in extreme environments without additional hardware burden.

Given its ease of application, taVNS could be seamlessly integrated into astronaut equipment, such as helmets (Figure 2) or head-mounted interfaces, ensuring accessibility during critical mission phases. This integration could allow on-demand cognitive and psychomotor optimization, particularly in high-risk scenarios such as docking maneuvers, extravehicular activities (EVAs), and deep-space navigation, where executive function and fine motor control are essential. By leveraging neuromodulation technology, astronauts could potentially counteract the adverse effects of microgravity on sensorimotor function, thereby improving operational safety and mission success.

taVNS could emerge as a key component of astronaut health and performance maintenance, offering a scalable, portable, and non-pharmacological countermeasure to the cognitive and physiological demands of space travel. By bridging the gap between space medicine and terrestrial applications, continued research on taVNS could drive advancements in performance optimization, mental health care, and cognitive rehabilitation, ultimately contributing to both space exploration and improvements in human health and efficiency across various high-demand environments.

## Data Availability

The original contributions presented in the study are included in the article/supplementary material, further inquiries can be directed to the corresponding author.
